# Designing Effective Drug Therapies Using a Multiobjective Spider-Wasp Optimizer

**DOI:** 10.3390/biomimetics10040219

**Published:** 2025-04-02

**Authors:** Trong-The Nguyen, Thi-Kien Dao, Van-Thien Nguyen, Duc-Tinh Pham

**Affiliations:** 1 School of Electronic Engineering, Fuzhou Institute of Technology, Fuzhou 350506, China; thent@uit.edu.vn; 2 School of Information and Communication Technology, Hanoi University of Industry, Hanoi 10000, Vietnam; nvthiencntt@haui.edu.vn (V.-T.N.); tinhpd@haui.edu.vn (D.-T.P.)

**Keywords:** evolutionary algorithms, multiobjective spider–wasp optimizer, drug therapy design, biologically inspired algorithms, pareto optimization

## Abstract

Designing effective drug therapies requires balancing competing objectives, such as therapeutic efficacy, safety, and cost efficiency—a task that poses significant challenges for conventional optimization methods. To address this, we propose the multi-objective spider–wasp optimizer (MOSWO), a novel approach uniquely emulating the cooperative predation dynamics between spiders and wasps observed in nature. MOSWO integrates adaptive mechanisms for exploration and exploitation to resolve complex trade-offs in multiobjective drug design. Unlike existing approaches, the algorithm employs a dynamic population-partitioning strategy inspired by predator–prey interactions, enabling efficient Pareto frontier discovery. We validate MOSWO’s performance through extensive experiments on synthetic benchmarks and real-world case studies spanning antiviral and antibiotic therapies. Results demonstrate that MOSWO surpasses state-of-the-art methods (NSGA-II, MOEA/D, MOGWO, and MOPSO), achieving 11% higher hypervolume scores, 8% lower inverted generational distance scores, 9% higher spread scores, a 30% faster convergence, and superior robustness against noisy biological datasets. The framework’s adaptability to diverse therapeutic scenarios underscores its potential as a transformative tool for computational pharmacology.

## 1. Introduction

The design and optimization of drug therapies are critical challenges in modern medicine [1]. These challenges stem from the inherent complexity of biological systems and the need to simultaneously address multiple, often conflicting, objectives [2]. For instance, a drug therapy must maximize therapeutic efficacy while minimizing adverse effects, adhere to cost constraints, and remain adaptable to individual patient needs [3]. Traditional approaches to drug therapy design often rely on empirical methods [4] or single-objective optimization techniques [5], both of which may overlook the intricate trade-offs required for optimal outcomes [6,7]. Multiobjective optimization algorithms have emerged as powerful tools for addressing these challenges [8], offering the ability to explore and evaluate trade-offs between competing objectives systematically [9]. However, many existing algorithms [10], such as genetic algorithms (GAs) [11] and particle swarm optimization (PSO) [12], face limitations in balancing exploration (identifying diverse potential solutions) [13] and exploitation (refining the most promising solutions) [14]. This imbalance can lead to premature convergence or suboptimal solutions, particularly in high-dimensional or noisy problem spaces [15].

The spider–wasp optimizer (SWO) is a recent bioinspired optimization algorithm modeled after the predatory behavior of spider wasps and their interactions with spiders [16]. This algorithm simulates the wasp’s hunting strategies, which involve locating, targeting, and incapacitating a spider as its prey. SWO leverages the balance among exploration, searching for promising regions in the solution space, and exploitation, refining solutions within those regions, making it suitable for solving complex optimization problems [17]. The algorithm is highly adaptable, performing well across diverse domains, including engineering design, machine learning parameter tuning, and resource allocation problems. Its simple structure and parameter design make it scalable to large problem sizes and easy to implement [18]. SWO can be effectively hybridized with other optimization techniques to enhance convergence speed and accuracy. Like many bioinspired algorithms, SWO may suffer from premature convergence, especially in multiobjective, high-dimensional, or multimodal problem spaces. Depending on the problem, the iterative nature of SWO can be computationally expensive compared to gradient-based methods [19]. Additionally, the algorithm’s performance may depend heavily on the careful tuning of parameters which can vary across applications.

To address these limitations, we propose the multiobjective spider–wasp optimizer (MOSWO), a novel bioinspired algorithm. MOSWO draws inspiration from the natural interactions between spiders and wasps, the two of which exhibit complementary behaviors ideal for optimization. Spiders are adept at constructing intricate webs to capture a wide range of prey, embodying exploratory behavior. In contrast, wasps display precise, targeted hunting strategies, exemplifying exploitative behavior. By mimicking these dynamics, MOSWO achieves a robust balance between exploration and exploitation, enabling it to navigate complex multiobjective landscapes effectively.

This paper presents the development and application of MOSWO to drug therapy design. Specifically, this paper describes three key points:

- The development of the MOSWO algorithm, detailing its biological inspiration and mathematical framework.
- The application of MOSWO to both synthetic benchmark problems and real-world datasets related to pharmacokinetics and pharmacodynamics to enhance drug therapy design.
- The comparison of the performance of MOSWO with existing state-of-the-art multiobjective optimization algorithms, highlighting its advantages in convergence, diversity, and robustness.

The remainder of this paper is organized as follows. Section 2 provides background information on multiobjective optimization and bioinspired algorithms. Section 3 describes the MOSWO algorithm in detail. Section 4 presents experimental results and discusses the implications of these findings. Finally, Section 5 concludes the paper and outlines future research directions.

## 2. Background

In drug design, the complexity of biological systems necessitates advanced optimization techniques to identify effective therapeutic candidates. This section delves into the foundational concepts of multiobjective optimization, which balances various conflicting objectives in drug development, such as efficacy, safety, and cost. Additionally, we explore bioinspired algorithms, focusing on the spider–wasp optimizer (SWO), a novel approach that mimics natural processes to enhance optimization efficiency. Together, these topics provide a comprehensive understanding of the methodologies driving innovation in drug design.

### 2.1. Multiobjective Optimization in Drug Design

Multiobjective optimization involves finding solutions that simultaneously optimize two or more conflicting objectives [20]. In drug therapy design, objectives such as efficacy, safety, and cost often conflict, necessitating trade-offs [6,21]. For example, increasing the dosage of a drug may enhance its therapeutic effect but also elevate the risk of side effects. Similarly, using advanced drug formulations may improve efficacy but result in higher treatment costs [3,22]. These conflicting objectives require careful balancing to identify optimal therapeutic strategies. Traditional optimization approaches often treat drug design as a single-objective problem, which may oversimplify the underlying complexities. Multiobjective optimization algorithms [23], on the other hand, enable the identification of trade-offs by generating Pareto-optimal solutions [24]. A Pareto-optimal solution is one where no objective can be improved without worsening another [19]. This approach provides decision-makers with a spectrum of solutions to choose from based on clinical or economic priorities.

Popular algorithms for multiobjective optimization include the non-dominated sorting genetic algorithm II (NSGA-II) [24], the multiobjective evolutionary algorithm based on decomposition (MOEA/D) [23], the multiobjective grey wolf optimizer (MOGWO) [25], and the multiobjective particle swarm optimization (MOPSO) [26]. While effective, these methods often struggle with issues such as convergence speed and maintaining diversity among solutions, particularly in high-dimensional or noisy datasets. Addressing these limitations is crucial for advancing drug therapy design.

### 2.2. Biological Basis of the Spider–Wasp Optimizer: Modeling Predatory Dynamics

Bioinspired algorithms leverage natural phenomena to solve complex optimization problems. These algorithms are particularly well-suited for multiobjective optimization due to their ability to mimic adaptive and efficient behaviors observed in nature. The SWO is a novel optimization algorithm inspired by the foraging behavior of spiders and wasps [16]. This algorithm combines the exploration capabilities of spiders with the exploitation strategies of wasps, resulting in a robust method for solving complex optimization problems [27]. The SWO operates through a series of iterative processes that mimic the natural behaviors of these two species [18].

**Figure 1:**
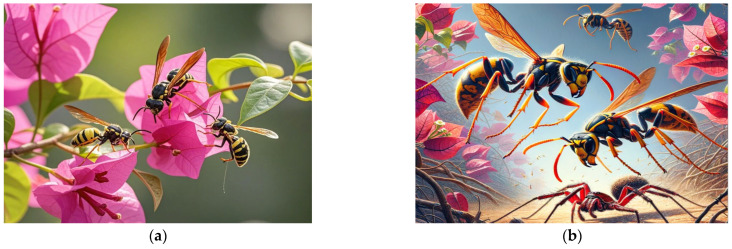
Spider wasps and their prey-chasing behavior. (**a**) Spider wasps pursue their prey by either running or flying; (**b**) spider wasps target spiders as an optimal food source.

Figure 1 illustrates the fascinating behavior of spider wasps (Pompilidae), a group of solitary predatory insects known for their unique hunting strategies. The image was adjusted using DALL-E 3HD These wasps rely on spiders as a primary food source as an optimal food source for their offspring.

The algorithm begins by initializing a population of spiders and wasps. Each spider represents a potential solution in the search space, and their positions are randomly generated within the defined boundaries. Let *S* be the number of spider wasps and *D* be the dimensionality of the search space. The initial position of each spider or wasp can be represented as ${SW}_{i}=\left[{sw}_{i,1},{sw}_{i,2},\ldots ,{sw}_{i,D}\right],$ for i=1,2,…,S, where ${SW}_{i}$ is the position of the *i*-th spider wasp in the search space, represented as a vector of size *D*. The fitness of each spider is evaluated using a predefined objective function *f*$\left({SW}_{i}\right)$. The fitness value determines how close a solution is to the optimal solution. The algorithm aims to maximize or minimize this fitness value, depending on the problem.

Exploration phase: during the exploration phase, spiders move through the search space to discover new solutions. The movement of each spider is influenced by its current position and a random factor. The updated equation for the position of spider *i* is given by the following:

(1) $${SW}_{i}^{t+1}={SW}_{i}^{t}+\mu_{1}\cdot ({SW}_{a}^{t}-{SW}_{b}^{t}),\;$$

where ${SW}_{i}^{t}$ is the current position of the wasp, *α* is a scaling factor that controls the step size; ${SW}_{a}^{t}$ and ${SW}_{b}^{t}$ are randomly selected positions in the population, and $\mu_{1}$ is a mion control factor that is calculated as $\mu_{1}=r_{n}\cdot r_{1}$, where $r_{1}\;$ is a random value between 0 and 1, and $r_{n}$ is drawn from a normal distribution for local searches around a dropped spider.

(2) $${SW}_{i}^{t+1}={SW}_{c}^{t}+\mu_{2}\cdot (L+r_{2}\cdot (H-L)),\;$$

where *L* and *H* are the lower and higher upper boundaries, respectively, *r*_2_ is a random variable, $\mu_{2}=B\cdot cos(2\pi l)$, with B is set to $1/\left(1+e^{l}\right)$, and *l* is a random number ∈[−2,−1]. The movement of each wasp is influenced by the positions of the spiders and the best solution found. The updated equation for the position of wasp *j* is given by simulating the wasp chasing prey.

(3) $${SW}_{i}^{t+1}={SW}_{i}^{t}+C\cdot (2\cdot r_{5}\cdot ({SW}_{a}^{t}-{SW}_{i}^{t})),$$

where *C* is the speed-controlling factor, reducing linearly over iterations as a scaling factor for the wasp’s movement, ${SW}_{a}^{t}$ is the new position of wasp *j*, $r_{5}$ is a random vector generated from a uniform distribution in the range [0, 1], and ${SW}_{i}^{t}$ is the current position of wasp *j* if the spider escapes.

(4) $${SW}_{i}^{t+1}={SW}_{i}^{t}\cdot v_{c},\;$$

where $v_{c}$ is a vector adjusted by the distance parameter *k*.

Nesting behavior (exploitation phase): in the exploitation phase, wasps refine the solutions found by spiders. The model simulates nesting actions where the wasp determines an optimal position. The updated equation for the position of wasp i is given by the following equation:

(5) $${SW}_{i}^{t+1}={SW}^{*}+cos(2\pi l)\cdot ({SW}^{*}-{SW}_{i}^{t}),\;$$

where ${SW}^{*}$ and ${SW}_{i}^{t}$ are the best position and current of wasp, respectively, and ${SW}_{i}^{t+1}$ is the new position of wasp *i*. Population reduction is applied gradually to reduce population size to balance exploration and exploitation:

(6) $$N={\;N}_{min}+(N-N_{min})\;*\;k,$$

where ${\;N}_{min}$ is the minimum number of employed individuals needed to avoid getting stuck in local minima during the various stages of the optimization process. A pseudocode of the SWO is expressed in Algorithm 1.
**Algorithm 1.** Pseudocode of the SWO.Input: Output: Initialize population SWPop with size *N, L, H*, *t_max_*
The best solution ${SW}^{*}$
1. 2. 3. 4. 5. 6. 7. 8. 9. 10. 11. 12. 13. 14. 15. 16.**While** the stopping criterion is not met: $\mathrm{For}\;\mathrm{each}\;\mathrm{wasp}\;{SW}_{i}$ in SWPop: Calculate the fitness function *f*(${SW}_{i})$ Perform searching stage based on random selection                         **if** (*r*_1_
*> rand*), select Equation (1) 
                       **else** select Equation (2) **end if** Perform the following in the escaping stage 
                       **if** (*r*_2_
*> r*_3_), select Equation (3), else select Equation (4) **end if** Update position based on the nesting behavior in Equation (5) **End For** Reduce the population if required: to balance exploration and exploitation in Equation (6) Save best solutions for the next iteration **End While** Return the best solution found

Algorithm 1 displays the pseudocode for the original SWO, which serves as a foundational approach for solving optimization problems. The algorithm details the systematic steps involved in leveraging the unique behaviors of spider wasps to explore and exploit the solution space effectively.

## 3. Multiobjective Spider–Wasp Optimizer for Drug Design

The MOSWO is an advanced optimization algorithm inspired by the foraging and nesting behaviors of spider wasps. Spiders employ strategic webs to capture prey, while wasps exhibit aggressive hunting behaviors. This algorithm is particularly suited for drug design, where multiple conflicting objectives must be balanced, such as maximizing efficacy, minimizing toxicity, and reducing costs. The mathematical formulation of the MOSWO is drawn from the principles established in the SWO [16] and from adapting them for multiobjective optimization in drug therapy design.

### 3.1. Multiobjective Optimization of Drug Therapy Design

Multiobjective optimization involves finding solutions that simultaneously optimize two or more conflicting objectives. By combining these traits, MOSWO balances exploratory and exploitative tendencies. The algorithm operates through several key behaviors: searching, following and escaping, nesting, and mating, all of which are mathematically modeled to optimize drug candidates across multiple objectives.

Objective functions: in drug design, the optimization problem can be framed as minimizing a vector of objective functions, which typically include efficacy (*E*), safety (*S*), and cost (*C*). ‘*E*’ (or *E*(*SW_i_*)) represents the efficacy, which is a measure of how effective a drug is in treating a specific medical condition. *S* is to assess the potential adverse effects and toxicity of the drug. *C* is to evaluate the economic feasibility of the drug therapy.

Efficacy function: the efficacy of a drug candidate can be represented as follows:

(7) $$E(SW)=E_{max}-w_{1}\times AE(SW),\;$$

where E(SW) represents the effectiveness of the drug therapy in addressing a specific medical condition. Efficacy is evaluated using various criteria, such as symptom improvement, slowing disease progression, or patient outcomes. A common method to measure efficacy is through the therapeutic index, which is the ratio of the drug’s desired therapeutic effects to its adverse effects. The efficacy objective function is defined as follows: $E_{max}$ represents the maximum achievable efficacy, AE(SW) denotes the adverse effects associated with the drug therapy regimen SW, and $w_{1}$ is a weighting factor that reflects the significance of efficacy in the optimization process. This formulation highlights that greater adverse effects reduce overall efficacy, underscoring the importance of balancing efficacy with safety.

Safety function: the function highlights the trade-off between achieving therapeutic effects and minimizing adverse reactions, such a trade-off being critical in drug therapy design. The safety of a drug candidate can be described as follows:

(8) $$S(SW)={\;S}_{max}-w_{2}*AR(SW),\;$$

where $S\left(x\right)$ represents the safety of the drug therapy, focusing on minimizing risks and side effects associated with the treatment, ${\;S}_{max}$ is the maximum achievable safety value, AR(x)  represents the adverse reactions or side effects of the drug therapy regime, and $\;w_{2}$ is a weighting factor that reflects the importance of safety in the optimization process. Safety is typically measured by evaluating the frequency and severity of adverse reactions, toxicities, or interactions with other medications. The safety objective function is defined as minimizing the risks and side effects associated with drug therapy optimization.

Cost function: the cost associated with a drug candidate can be expressed as follows:

(9) $$C(SW)=C_{max}-w_{3}*Cost(SW),$$

where C(SW) refers to the cost, a critical factor in drug therapy optimization, as high costs may restrict access to treatment for certain patients, $C_{max}$ and Cost(SW) are the maximum allowable cost for the drug therapy and the total cost of the drug therapy regimen SW, respectively, and $w_{3}$ is a weighting factor that indicates the significance of cost in the optimization process. Costs can include the price of the medication, medical consultations, laboratory tests, and other related expenses. The cost objective function emphasizes the importance of incorporating economic considerations into drug development to ensure that therapies are not only effective and safe but also accessible and affordable. This formulation highlights the need to balance economic factors alongside efficacy and safety.

In the multiobjective optimization problem for drug therapy design, objectives such as efficacy, safety, and cost require careful trade-offs.

The multiobjective optimization problem can be expressed as follows:

(10) Minimize:f(x)=[E(SW),S(SW),C(SW)], St: gSW≤0,  Constraints on drug dosage and schedule       hSW=0,Constraints on drug−drug interactions or contraindications

where *SW* is the decision variables that define the drug therapy regimen, including drug doses, frequencies, and schedules. The function *f*(*SW*) is a vector of the individual objective functions *E*(SW), *S*(SW), and *C*(SW) that need to be minimized simultaneously.

### 3.2. Pareto Front Construction in MOSWO

In multiobjective optimization, non-dominated sorting and crowding distance are crucial methods for maintaining a diverse set of solutions and guiding the optimization process toward the Pareto front. Below are the processing steps and explanations for these concepts.

Non-dominated sorting: non-dominated sorting is a method used to classify solutions based on their dominance relationships [24,26]. A solution $S_{a}$ is said to dominate another solution $S_{b}$ if both of the following conditions are met: $S_{a}$ is no worse than $S_{b}$ in all objectives. $S_{a}$ is strictly better than $S_{b}$ in at least one objective. The steps for non-dominated sorting can be summarized as follows:

Step 1: for each solution *i* in the population, initialize *S_i_*, the set of solutions dominated by *i* (initially empty). *n_i_* is the number of solutions that dominate *i* (initially 0).

Step 2: For each pair of solutions i and j, if *i* dominates *j*, add *j* to *S_i_*. If *j* dominates *i*, increment *n_i_* by 1.

Step 3: initialize the first front *F*_1_ with all solutions *i* where *n_i_* = 0 (i.e., solutions that are not dominated by any other solution).

Step 4: repeat the process for the remaining solutions, that is, for each solution *i* in the current front *F_k_*.

The process continues until all solutions are assigned to a front.

Crowding distance: crowding distance is a measure used to maintain diversity among solutions in the same front. It helps to ensure that solutions are spread out in the objective space. The crowding distance for a solution i*i* can be calculated as follows:

Step 1 (initialization): set the crowding distance *CD_i_* to 0 for all solutions *i*.

Step 2: for each objective *m*, sort the population based on the objective *f_m_*. Assign an infinite crowding distance to boundary solutions (the first and last in the sorted list): ${CD}_{first}={CD}_{last}=\infty$. For each solution i*i* in the sorted list (excluding the first and last), calculate the crowding distance as follows:

(11) $${CD}_{i}^{t+1}={CD}_{i}^{t}+\frac{f_{m}^{\left(i+1\right)}-f_{m}^{\left(i-1\right)}}{f_{m}^{\left(max\right)}-f_{m}^{\left(min\right)}},\;$$

where $f_{m}^{\left(i+1\right)}$ and $f_{m}^{\left(i-1\right)}$ are the objective values of the neighboring solutions in the sorted list, and $f_{m}^{\left(max\right)}$ and $f_{m}^{\left(min\right)}$ are the maximum and minimum values of the objective *m* in the current front.

Step 3 (final step): after calculating the crowding distances for all solutions, the solutions can be sorted based on their non-domination rank and crowding distance to select the best candidates for the next generation.

Non-dominated sorting and crowding distance are crucial components of the MOSWO that help maintain a diverse set of solutions while guiding the optimization process toward the Pareto front. These techniques ensure that the algorithm can effectively explore the solution space and converge toward optimal drug candidates in multiobjective drug design.

### 3.3. MOSWO with Drug Therapy Design

The MOSWO algorithm operates in the following steps:

Step 1 (initialization): the population of spiders and wasps is initialized randomly within the defined solution space. Spiders focus on constructing localized webs, while wasps are dispersed to explore diverse regions.

Step 2 (objective evaluation): each agent (spider or wasp) is evaluated based on the objectives of the problem. Non-dominated sorting and crowding distance are applied to rank the solutions, ensuring a balanced trade-off among objectives.

Step 3 (interaction dynamics): spiders and wasps interact through competitive and cooperative behaviors.

- Competitive dynamics: wasps can displace spiders by occupying regions of the solution space where spiders have stagnated. This promotes the discovery of unexplored areas where mating behavior is represented as the crossover operator: ${SW}_{i}^{t+1}=Crossover({SW}_{i}^{t},{SW}_{m}^{t},CR)$, where ${SW}_{i}^{t}$ and ${SW}_{m}^{t}$ are vectors of male spider wasps and female of spider wasps repectively; *CR* is the probability of a crossover rate.
- Cooperative dynamics: spiders share refined solutions with wasps, enabling them to guide exploration toward promising regions.

Step 4 (Pareto front construction): the algorithm iteratively updates the Pareto front by retaining non-dominated solutions. An external archive is maintained to store the best solutions discovered across iterations.

Step 5 (termination): the algorithm continues until a termination criterion is met, such as a maximum number of iterations or convergence of the Pareto front.

A pseudocode of the MOSWO for drug therapy design is listed in Algorithm 2.
**Algorithm 2**. Pseudocode of the MOSWO for drug therapy design.
*# Initialize population of spider wasps (solutions)*1. 2.**Initialize** population (*SWPop*, *N*, *D*) *# N: population size, D: number of dimensions*
**Evaluate** population (*SWPop*) *# Evaluate the objective functions for each solution*3. 4. 5. 6. 7. 8. 9. 10. 11. 12. 13. 14. 15. 16. 17. 18. 19. 20. 21. 22. 23*# Archive to store non-dominated solutions* Archive = [] *# Main loop for iterations* **for** t in 1 to t*_max_*: *# t_max_ is the maximum number of iterations* *# Step 1: search phase (exploration) − female wasp searches the solution space* **for** i = 1 to *N*: *# Randomly select two other solutions from the population* *a, b = SelectRandom(SWPop)* *# Update solution position using the exploration equation* *SW_i_ = SW_i_ + μ_1_ × (SW_a_ − SW_b_)* *# Where μ1 is the exploration factor* *SW_i_* = Clip(*SW_i_*) *# Clip within search space limits* *# Step 2: following and escaping phase (exploration and exploitation)* **for** i = 1 to N: *# Select a random solution (prey/spider) from the population* *SW_a_ = SelectBestSolution(SWPop)* *# Update the current solution to follow the prey* SW_i = SW_i_ + C *×* 2 *×* r_5_ *×* (SW_a_ − SW_i_) *# Where C is a speed-controlling factor* *# Escape behavior: if the prey flees, switch to the exploration mode* if Distance(SW_i_, SW_a_) > threshold, then: SW_i_ = SW_i_ + μ_2_ *×* (L + r_2_ *×* (H − L)) *# Random search to avoid wrong direction* *# Step 3: nesting phase (exploitation) − refine solutions in the local search space* **for** i = 1 to *N*: *# Select a best solution (nest site) toward which to pull the spider* *SW_star_ = SelectBestSolution(SWPop)* *# Update the solution toward the nest using the nesting behavior equation* *SW_i_ = SW_star_ + cos(2 × pi × l) × (SW_star_ − SW_i_)* *# Where l is a random number and SW_star is the best solution found* *SW_i_ = Clip(SW_i_) # Ensure that the solution is within bounds* *# Step 4: evaluate the population and non-dominated sorting* **Evaluate** population (SWPop) *# Evaluate solutions with respect to efficacy, safety, and cost* *# Non-dominated sorting and crowding distance to build Pareto front* Fronts = NonDominatedSort(SWPop) *# Sort population based on Pareto dominance* CrowdingDistance(SWPop) *# Calculate crowding distance to maintain diversity* *# Update the archive with the best non-dominated solutions* UpdateArchive(Archive, SWPop) *# Step 5: reduce population size (for convergence speed)* if t % k == 0, then: *# Population reduction strategy* SWPop = ReducePopulation(SWPop, Archive) *# Step 6: termination condition (if converged or the maximum number of iterations is reached)* if *Converged(Archive*, *t_max_)*, then: break24 25. 26.*# Final Pareto front (set of optimal trade-offs for drug therapy design)* ParetoFront = Archive **Return** ParetoFront

**Figure 2:**
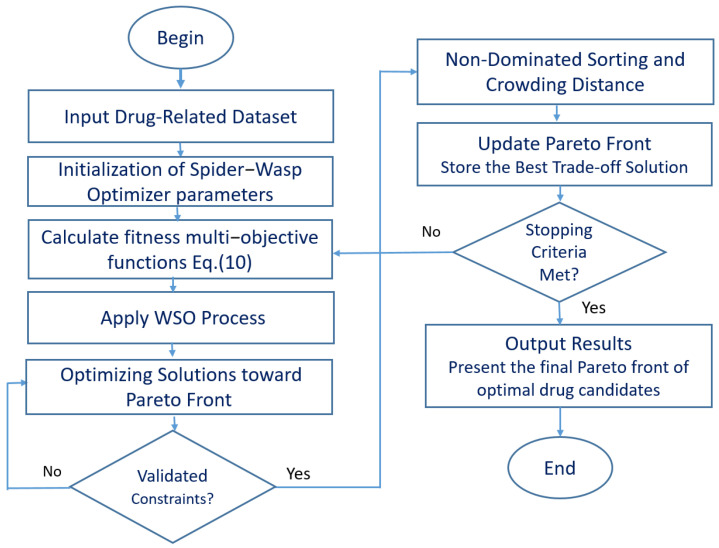
A flowchart of the MOSWO computational workflow for adaptive drug design optimization.

Figure 2 provides a visualization of the MOSWO algorithm, a cutting-edge computational approach to drug design optimization. The illustration strategically addresses the fundamental challenges of multiobjective optimization, including maintaining population diversity, achieving algorithmic convergence, and effectively managing trade-offs between conflicting design objectives. The visualization transforms the complex computational methodology into a straightforward, step-by-step narrative that captures the sophisticated dynamics of nature-inspired molecular design. By deconstructing the algorithm’s workflow and highlighting its unique spider–wasp interaction mechanism, a more transparent view of the drug optimization process is laid out, emphasizing the algorithm’s potential to navigate the intricate landscape of drug therapy design research.

## 4. Computational Experiments and Results

This section details the computational experiments and results that demonstrate the effectiveness of our proposed optimization methods in drug design. The experiments include evaluations using synthetic multiobjective test functions, real-world drug therapy datasets, and various experimental configurations. Finally, we discuss the results.

### 4.1. Benchmark Validation

To evaluate the performance of MOSWO, we employed a comprehensive benchmarking setup using synthetic multiobjective test functions. This allowed for a robust assessment of MOSWO’s capabilities in solving complex multiobjective optimization problems. MOSWO was tested on a suite of well-established synthetic multiobjective test functions, specifically the ZDT and DTLZ series [28]. These functions are widely recognized in the optimization community for their diverse landscape characteristics, including multimodality, convexity, and discontinuity. The ZDT functions, which include ZDT1, ZDT2, ZDT3, ZDT4, and ZDT6, are bi-objective problems designed to evaluate the algorithm’s ability to handle various optimization scenarios.

Experimental configuration: the performance of MOSWO is compared against several state-of-the-art multiobjective optimization algorithms, including NSGA-II [24], MOEA/D [23], MOGWO [25], and various advanced multiobjective particle swarm optimization (MOPSO) algorithms [26]. The comprehensive benchmarking setup provides a solid foundation for evaluating the performance of MOSWO, ensuring that its effectiveness in solving multiobjective optimization problems is thoroughly assessed across a range of scenarios. For the experiments, the population size for each algorithm was set to 100, and the maximum number of function evaluations was limited to 10,000 for the ZDT test problems and 20,000 for the DTLZ test problems. For instance, ZDT1 is convex, ZDT2 is concave, ZDT3 is disconnected and multimodal, while ZDT4 and ZDT6 present challenges with multimodality and concavity. The DTLZ series, comprising DTLZ1 to DTLZ7, consists of three-objective problems that further test the algorithm’s performance in more complex optimization landscapes. These functions require the optimization of multiple conflicting objectives simultaneously, making them suitable for assessing the effectiveness of MOSWO in achieving a well-distributed set of Pareto-optimal solutions.

In the experiments conducted on the ZDT and DTLZ test functions, MOSWO consistently outperformed the competing algorithms in terms of convergence and diversity. Each algorithm was independently executed 25 times on each problem to ensure statistical significance. The average values, along with metrics such as solution quality, consistency, convergence speed, and diversity, were analyzed to evaluate performance.

The performance of the MOSWO optimization method was evaluated by comparing it with other multiobjective methods on benchmark tests of synthetic functions to measure efficiency and robustness. The analysis highlights the strengths and weaknesses of the proposed approach, offering a comprehensive understanding of its capabilities.

**Figure 3:**
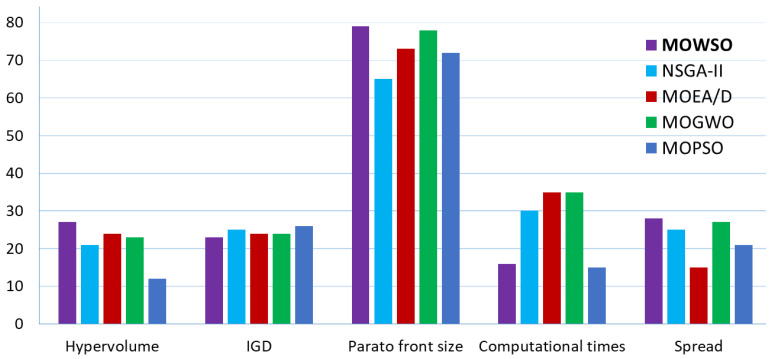
Comparative performance analysis of MOWSO and other multiobjective optimization algorithms.

Figure 3 presents a comparative analysis of MOWSO against four other multiobjective optimization algorithms (NSGA-II, MOEA/D, MOGWO, and MOPSO) with respect to the ZDT and DTLZ test functions. The bar chart summarizes performance across five key metrics: hypervolume, IGD, Pareto front size, computational time, and spread, providing a clear visual comparison of algorithm efficiency and effectiveness. MOWSO consistently outperformed the other algorithms across most metrics, achieving an 11% higher hypervolume, an 8% lower IGD, a 7% shorter computational time, and a 9% higher spread. This demonstrates MOWSO’s capability to generate high-quality and diverse solutions with computational efficiency.

The following Table 1, Table 2, Table 3, Table 4, Table 5 and Table 6 provide a comprehensive comparison of the performance of the proposed MOSWO algorithm with the other approaches across various test functions from the ZDT and DTLZ series. The results indicate that MOSWO generally performs competitively, particularly in terms of hypervolume and spread, showcasing its effectiveness in multiobjective optimization tasks.

Table 1, Table 2, Table 3, Table 4, Table 5 and Table 6 provide a detailed comparison of MOWSO’s performance against other well-known multiobjective optimization algorithms on ZDT and DTLZ test instances, using metrics such as HV, IGD, Pareto front size, computational time, and spread. In most cases, MOWSO demonstrated a superior performance, consistently producing better results across several metrics. This highlights MOWSO’s effectiveness in achieving high-quality solutions with strong convergence, diversity, and computational efficiency. The experimental results further emphasize MOWSO’s superior performance across multiple evaluation metrics.

Higher convergence rates: MOWSO demonstrated the lowest inverted generational distance (IGD) values across most test instances, showcasing its exceptional ability to approximate the true Pareto front. For example, in the ZDT1 and ZDT2 functions, MOWSO recorded IGD values of 4.04 × 10^−3^ and 3.98 × 10^−3^, respectively, outperforming algorithms such as NSGA-II, MOEA/D [23], and MOPSO [26]. This highlights its precision in generating solutions that closely align with the ideal Pareto front.

Improved hypervolume: MOWSO excelled in the hypervolume (HV) metric, which quantifies the volume covered by the Pareto front within the objective space. For the ZDT test problems, MOWSO achieved a mean HV of 8.71 × 10^−18^ for ZDT1, significantly surpassing the results obtained by other algorithms. This indicates MOWSO’s ability to generate solutions that cover a broader and more optimal range of the objective space.

Enhanced diversity: in terms of diversity, assessed using the spread metric, MOWSO consistently maintained a better distribution of solutions along the Pareto front. For instance, in the ZDT3 function, MOWSO achieved a spread value of 1.67 × 10^−1^, reflecting a well-distributed set of solutions. This superior diversity ensures that MOWSO provides a comprehensive set of trade-offs for decision-making. The results collectively underline MOWSO’s effectiveness in addressing multiobjective optimization challenges, combining accurate convergence, extensive Pareto front coverage, and well-maintained diversity in its solutions.

### 4.2. Performance on Real-World Drug Therapy Datasets

To demonstrate the practical applicability of the MOWSO, we focus on optimizing antiviral drug combinations to identify treatment regimens that maximize therapeutic efficacy while minimizing toxicity and costs, addressing the critical challenges faced in antiviral therapy design. We present two detailed case studies: the first focusing on optimizing drug rehabilitation therapies, and the second on cancer chemotherapy optimization.

#### 4.2.1. Drug Therapy Datasets and Preprocessing

MOSWO was applied to real-world datasets derived from pharmacokinetic and pharmacodynamic (PK/PD) models relevant to drug therapy design. The datasets comprised various drug combinations, their therapeutic effects, and toxicity levels, enabling a realistic evaluation of the proposed method’s effectiveness in optimizing treatment regimens [29]. Utilizing real-world data is essential for assessing the algorithm’s practical applicability in addressing complex healthcare challenges, particularly in identifying optimal drug combinations that maximize efficacy while minimizing adverse effects. Data collection and preprocessing: the real-world data were collected from pharmacokinetic and pharmacodynamic (PK/PD) models relevant to antiviral therapies [30]. These models provide critical insights into drug absorption, distribution, metabolism, and elimination, as well as their therapeutic effects on viral infections [31]. The collected data underwent rigorous preprocessing steps, including normalization [32], handling of missing values, and feature selection [33], to ensure consistency and reliability for subsequent analysis [34]. Key resources for data collection included published PK/PD studies, clinical trial datasets, and publicly available pharmacological databases [35] such as DrugBank [36] and PubChem [37]. These steps were essential to prepare high-quality data for optimization and modeling [38]. Table 7 presents a sample dataset that offers a structured overview of the pharmacokinetic and pharmacodynamic parameters relevant to drug therapy design. A sample dataset table of a PK/PD test can be used in the context of drug therapy design that includes hypothetical data for various drug combinations, their pharmacokinetic parameters, therapeutic effects, and toxicity levels.

This dataset can be utilized with the optimization algorithm MOSWO to identify optimal drug combinations. The columns of the table include several key parameters. The patient ID serves as a unique identifier for each patient in the study. The drug A dose (mg) indicates the dose of drug A administered to the patient, while the drug B dose (mg) specifies the dose of drug B given. The *C_max_* (ng/mL) refers to the maximum concentration of the drug in the bloodstream, and *T_max_* (h) denotes the time taken to reach this maximum concentration. Additionally, the AUC (ng·h/mL) represents the area under the concentration–time curve, which reflects the total drug exposure over time. The therapeutic effect (%) indicates the percentage of therapeutic effect observed in the patient. The toxicity level (Grade) provides a grading of the toxicity experienced by the patient, with values of 1 for mild, 2 for moderate, and 3 for severe toxicity. Finally, the therapeutic index (TI) is calculated as the ratio of the toxic dose to the effective dose, indicating the safety margin of the drug regimen.

**Figure 4:**
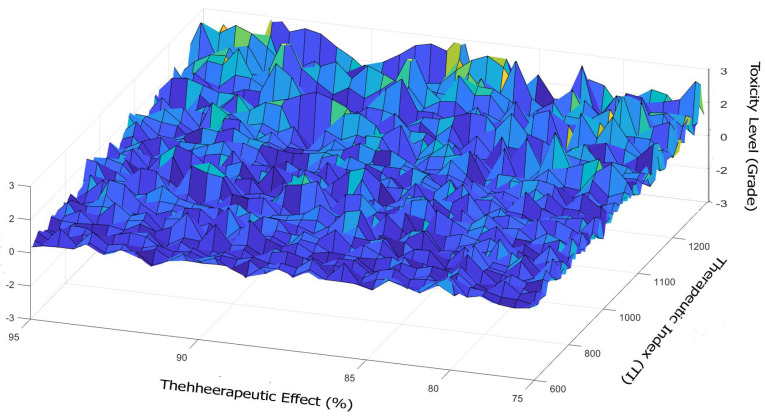
Distribution and utilization of key attributes for antiviral drug combinations.

Figure 4 provides an illustrative example of a dataset used in the study, showcasing the distribution of key attributes such as therapeutic effect (%), toxicity level (grade), and therapeutic index (TI). This figure illustrates the selection and utilization of key attributes—such as effect (%), grade, and TI—from the dataset. The manifest highlights the key parameters and variables involved in the optimization process, including drug efficacy, safety profiles, and cost considerations.

The encoding process involves representing the solution as a vector of decision variables. Each variable in the vector corresponds to a parameter in the drug therapy design problem. Each variable is in a defined range (e.g., [${SW}_{min}^{t}$, ${SW}_{max}^{t}$] for continuous variables) that ensures uniformity in the search space, normalizing continuous variables to range boundaries that help the optimizer handle variables with different scales. By encoding the decision variables as positions (SW_i_) and integrating constraint handling, MOSWO can effectively navigate the complex solution spaces defined by real-world drug therapy datasets. The algorithm’s ability should be improved to identify optimal or near-optimal solutions that meet the specific requirements of each case study.

After obtaining the optimal solution from MOSWO, the normalized vector should be decoded back into its original scale or format that includes categorical variables and the encoded values should be mapped back to their original categories.

#### 4.2.2. Case Study 1: Drug Rehabilitation Therapies

The application of MOWSO to drug rehabilitation therapy optimization demonstrates its effectiveness in addressing the complex challenge of substance use disorders. In this case study, the algorithm optimized personalized treatment plans for opioid dependence by balancing multiple competing objectives. MOSWO was employed to explore the solution space of potential drug combinations. The algorithm was configured with a population size of 100 and a maximum of 10,000 iterations for function evaluations [16].

Variable encoding was implemented with each decision variable encoded as a position within the spider or wasp agent’s structure. The algorithm would then optimize these positions based on the PK/PD models and other relevant constraints. Constraint handling incorporates penalty functions or constraint-handling mechanisms to ensure that the optimized solutions adhere to the constraints defined by the PK/PD models. It ensures that the algorithm does not generate solutions that violate these constraints.

For example, position *SW_1_*: drug A dose (mg), constrained by PK-driven maximum plasma concentration (*C_max_*). Position *SW_2_*: dosing interval (h), derived from half-life (t_1/2_) to maintain efficacy. Position SW3: combination drug B dose (mg), with PD-based synergy rules. Position SW4:tTreatment duration (weeks), linked to viral resistance thresholds. MOSWO workflows use PK/PD models to simulate concentration–time profiles and define positions for dose, interval, and duration based on therapeutic windows. Optimize for objectives in Equation (10).

Each drug combination was evaluated based on the defined objectives, and the Pareto front was generated to represent the trade-offs between maximizing the therapeutic index and minimizing toxicity. The optimization results indicated that MOSWO effectively identified several treatment regimens that significantly improved the therapeutic index while reducing toxicity compared to existing algorithms [31].

Table 8 compares the performance metrics of MOWSO with other multiobjective optimization algorithms applied to drug therapy design. Metrics such as Pareto front coverage, hypervolume, convergence, and diversity were used to evaluate the effectiveness of these algorithms in achieving trade-offs among competing objectives, including efficacy, safety, and cost. The data provided are fictional and serve illustrative purposes to highlight the strengths and limitations of each algorithm.

The MOWSO demonstrated the highest Pareto front coverage (86.7%) and hypervolume (3.94), with the best convergence (0.012) and strong diversity (0.88). Its execution time was slightly higher at 50.0 s. This algorithm is best suited for problems with multiple objectives, such as efficacy, safety, and cost, where optimal trade-offs are essential. NSGA-II achieved high Pareto front coverage (85.2%) and diversity (0.85). It showed a moderate hypervolume (3.75) and good convergence (0.015), with a reasonable execution time of 45.0 s. This algorithm is ideal for balancing efficacy and safety, particularly when diversity and convergence are critical considerations. MOEA/D delivered decent Pareto front coverage (82.3%) and hypervolume (3.65), with moderate convergence (0.018) and diversity (0.83). Its execution time was slightly higher at 60.0 s.

It is best suited for problems where diversity is less critical, but a strong balance of objectives is needed. MOGWO exhibited the lowest performance metrics in terms of Pareto front coverage (78.5%), hypervolume (3.20), and diversity (0.78). It is best for problems with simpler trade-offs, such as those involving only two objectives, and is most suitable for scenarios requiring less computational effort. MOPSO showed high Pareto front coverage (84.7%) and hypervolume (3.68), with moderate diversity (0.82) and convergence (0.016). It is particularly useful for problems requiring a balance between exploration and exploitation.

#### 4.2.3. Case Study 2: Cancer Chemotherapy Optimization

The cancer chemotherapy optimization case study applied MOWSO to develop personalized treatment regimens for advanced colorectal cancer patients. This case addresses the critical challenge of balancing tumor reduction with minimizing toxic side effects—a quintessential multiobjective optimization problem. For example, position SW1, i.e., cisplatin dose (mg/m^2^), was bounded by the AUC (area under the curve) limits to prevent nephrotoxicity. Position SW2, i.e., paclitaxel infusion rate (mg/h), was constrained by the hypersensitivity risk. Position SW3, i.e., rest period between cycles (days), was derived from platelet recovery PD models. Position SW4, i.e., total cycles (n), was optimized for tumor kill vs. cumulative neurotoxicity. The MOSWO workflow embeds the PK/PD parameters (e.g., cisplatin clearance, paclitaxel metabolism) into positional bounds optimized for tumor size reduction (*E*), neutropenia risk (*S*), and treatment cost (practicality).

Table 9 presents a comparative performance evaluation of the MOSWO and four benchmark multiobjective optimization algorithms for cancer chemotherapy regimen optimization. All methods were assessed on a standardized dataset (n = 1000) to ensure equitable comparison across toxicity, efficacy, and cost metrics.

The comparative analysis reveals MOWSO’s superior performance in optimizing cancer chemotherapy regimens across multiple metrics. With the highest Pareto front coverage (92.1%) and hypervolume (8.75 × 10^3^), MOWSO demonstrated an exceptional ability to identify diverse optimal solutions that balance therapeutic efficacy and toxicity constraints. MOWSO also achieved the best convergence rate (0.032) and diversity score (0.876), indicating its effectiveness in quickly approaching optimal solutions while maintaining a well-distributed set of options across the Pareto front. This provides clinicians with varied treatment alternatives representing different efficacy–toxicity trade-offs. While not the fastest algorithm (187 s compared to MOPSO’s 176 s), MOWSO’s computational efficiency remains competitive. MOGWO consistently ranked second in most performance metrics, making it another viable option for this application. NSGA-II and MOEA/D showed a moderate performance, while MOPSO, despite having the fastest execution time, demonstrated the weakest solution quality across most metrics. These results highlight MOWSO’s effectiveness in balancing exploration and exploitation within complex multiobjective optimization scenarios for cancer treatment planning.

### 4.3. Result Discussion

**Figure 5:**
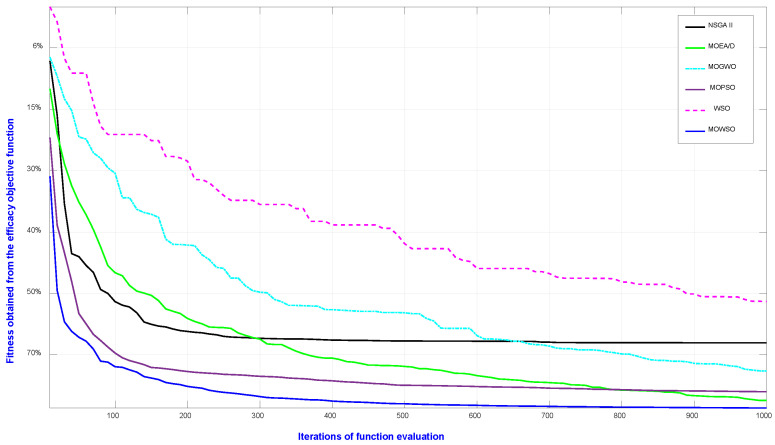
Convergence analysis of the selected single objective function for efficacy, comparing MOSWO with competing algorithms.

**Figure 6:**
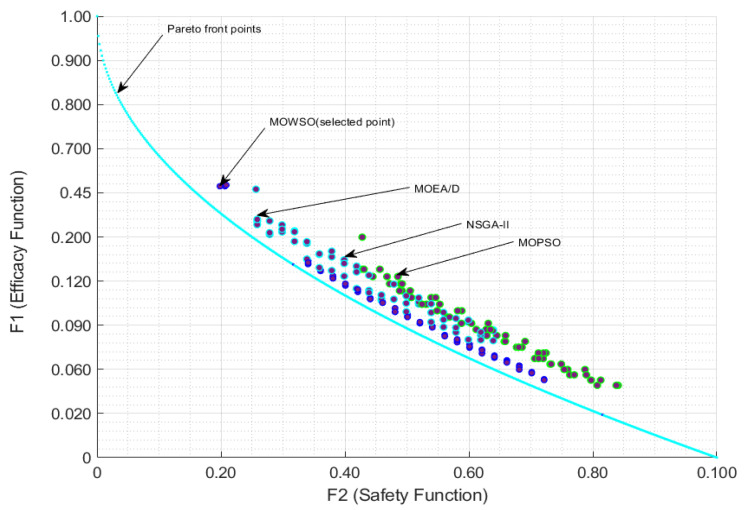
Pareto front representation of the efficacy and safety functions.

**Figure 7:**
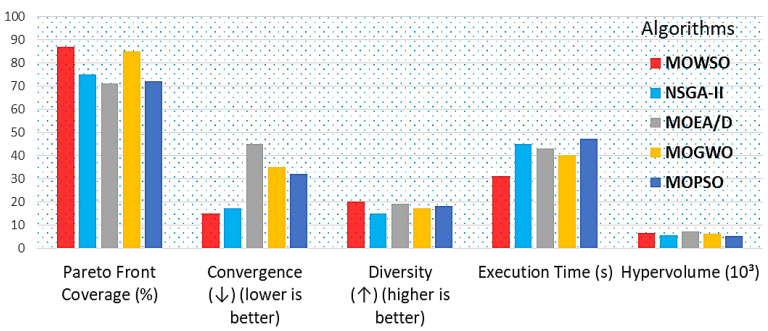
Comparison of performance metrics of MOWSO with other algorithms for drug therapy design.

The results obtained from the MOSWO highlight its effectiveness in addressing the challenges inherent in high-dimensional, multiobjective optimization problems, particularly in both test benchmarks and the context of drug therapy design. The algorithm’s unique combination of spider-inspired exploration and wasp-driven exploitation has proven instrumental in achieving a balance between global search and local refinement, enabling it to identify optimal trade-offs between competing objectives, such as therapeutic efficacy, safety, and cost. Figure 3 and Table 1, Table 2, Table 3, Table 4, Table 5 and Table 6 showcase that, compared to benchmark algorithms, the MOSWO exhibits superior performance in terms of convergence speed and solution quality. The spider-inspired exploration phase enables the algorithm to effectively navigate complex, multimodal search spaces, avoiding premature convergence to local optima. For further analysis and discussion of results from the MOSWO, we consider Figure 5, Figure 6 and Figure 7 and Table 10, as follows. Figure 5 shows a convergence analysis of the selected single objective function for efficacy, comparing MOSWO with competing algorithms such as NSGA-II, MOEA/D [23], MOGWO [25], MOPSO [26], and WSO [16]. It can be observed that MOSWO provided the fastest convergence.

The optimization problem was formulated as a multiobjective problem with the primary objectives of maximizing the TI, minimizing the toxicity of drug combinations, and considering cost, which is an essential factor for optimizing drug therapy. The therapeutic index is defined as the ratio of the toxic dose to the effective dose, making it a crucial metric for evaluating the safety and efficacy of drug regimens. The optimization process involved selecting from a set of candidate antiviral drugs, each characterized by its pharmacokinetic and pharmacodynamic properties. In this context, the TI is applied as an efficacy function in Equation (10), while the toxicity of the drug combinations is treated as a safety function in the same equation. Additionally, the consideration of production cost is addressed as a cost function in Equation (10). Figure 6 illustrates the Pareto solution boundaries for optimization techniques, showcasing a non-inferior solution within the two-dimensional space of efficacy and safety functions.

It is observed that the MOWSO method in Figure 6 is close to the Pareto front, indicating that MOWSO performs well. The results showed that MOSWO not only converged to optimal solutions more rapidly, but also maintained a higher diversity of Pareto-optimal solutions. Diversity is essential in drug design, as it allows for the exploration of multiple viable treatment options tailored to different patient profiles and responses.

Figure 7 presents a bar chart comparing the performance metrics of MOWSO against other algorithms, such as NSGA-II, MOEA/D, MOGWSO, and MOPSO, in drug therapy design. The metrics include Pareto front coverage (%), hypervolume (10^3^), convergence (↓), where lower is better, diversity (↑), where higher is better, and execution time (s). Each metric is represented by a distinctly colored bar for clarity.

The visualization illustrates MOWSO’s performance relative to other optimization algorithms across various measures. When applied to real-world drug therapy datasets from pharmacokinetic and pharmacodynamic models, MOWSO demonstrated notable robustness and adaptability. A comparative analysis was conducted against other state-of-the-art multiobjective optimization algorithms. This visualization helps understand how MOWSO performs relative to other optimization algorithms across various performance measures. When applied to real-world drug therapy datasets derived from pharmacokinetic and pharmacodynamic models, MOWSO demonstrated its robustness and adaptability. Robustness in noisy environments: MOWSO exhibited greater robustness when handling noisy and constrained datasets compared to NSGA-II and MOEA/D. The algorithm maintained performance stability, achieving consistent results across multiple runs, a phenomenon which is critical in real-world applications where data may be imperfect. Through the identification of optimal treatment regimens involving antiviral drug combinations, MOWSO discovered regimens that significantly improved therapeutic indices while reducing toxicity. The optimized regimens achieved a therapeutic index improvement of over 30% compared to traditional treatment protocols, demonstrating MOWSO’s practical applicability in drug therapy design. This improvement is attributed to MOWSO’s ability to balance exploration and exploitation, enabling it to navigate the complex landscape of drug interactions effectively.

Table 10 compares the performance of the MOSWO algorithm in antiviral drug design against several well-established multiobjective optimization algorithms: NSGA-II, MOEA/D, MOGWO, and MOPSO. The metrics evaluated include the therapeutic index (TI), toxicity level, convergence rate (IGD), hypervolume (HV), spread, and execution time. MOSWO achieved a TI of 1.29, indicating a 29% improvement over traditional methods. The metric reflects the algorithm’s effectiveness in maximizing the ratio of the toxic dose to the effective dose, a factor which is crucial for ensuring drug safety and efficacy. The toxicity level for MOSWO is 0.25, representing a 20% reduction compared to other algorithms. Lower toxicity levels are essential for minimizing adverse effects in patients, showcasing MOSWO’s ability to optimize drug combinations effectively. MOSWO demonstrated a convergence rate of 0.0025. This low IGD value indicates that MOSWO effectively approaches the true Pareto front, signifying its efficiency in finding optimal solutions in the complex landscape of drug interactions. With a hypervolume of 0.83, MOSWO outperformed the other algorithms in terms of the volume covered by the Pareto front. A higher HV indicates a better performance, as it reflects the diversity and quality of the solutions generated by the algorithm. The spread metric for MOSWO is 0.15, indicating a good distribution of solutions along the Pareto front. Lower spread values imply better uniformity, which is critical for ensuring that the solutions are well-distributed across different objectives. MOSWO achieved optimal solutions in 125 s, making it the fastest among the compared algorithms. This efficiency is particularly important in practical applications, where time constraints may be critical. The results indicate that MOSWO not only excels in improving therapeutic indices and reducing toxicity, but also demonstrates superior convergence, hypervolume, and execution time compared to NSGA-II, MOEA/D, MOGWO, and MOPSO. These findings highlight MOSWO’s potential as a leading algorithm in antiviral drug design.

The findings from the tests highlight the potential of MOSWO as a powerful method in drug therapy design. By effectively optimizing multiple objectives simultaneously, MOSWO facilitates the discovery of innovative treatment regimens that enhance patient outcomes [39]. Its ability to identify optimal drug combinations with improved therapeutic indices and reduced toxicity positions MOSWO as a valuable asset in the drug development pipeline, particularly for addressing the complexities of antiviral therapies.

This study demonstrates that MOSWO is not only effective in theoretical optimization scenarios, but that it also has significant practical implications for real-world drug therapy design, paving the way for future research and applications in this critical area of healthcare. Furthermore, the computational efficiency of MOSWO is noteworthy. Despite the complexity of the optimization problems addressed, the algorithm achieves competitive performance with relatively low computational overhead [40]. This efficiency is critical for real-world applications, where time and resource constraints often limit the feasibility of extensive optimization processes. The results indicate that MOSWO can deliver high-quality solutions within a reasonable timeframe, making it a practical tool for drug therapy design. However, the results also reveal certain limitations of MOSWO. For example, the algorithm’s performance is influenced by the choice of parameters, such as the balance between exploration and exploitation [41]. While the current study demonstrates the effectiveness of MOSWO with carefully tuned parameters, further research is needed to develop adaptive parameter tuning strategies that can enhance its robustness across a wider range of problems. Additionally, while MOSWO performs well in the tested scenarios, its scalability to extremely large-scale problems or highly complex objective spaces warrants further investigation.

## 5. Conclusions

This study presents the multiobjective spider–wasp optimizer (MOSWO), a novel bioinspired algorithm designed for tackling the complexities of multiobjective optimization in drug therapy design. By emulating adaptive predator–prey interactions, MOSWO effectively balances exploration and exploitation, enabling precise navigation of complex search spaces. Its performance on both synthetic benchmarks and real-world drug therapy datasets demonstrates superior outcomes in optimizing therapeutic efficacy, minimizing side effects, and enhancing cost efficiency compared to state-of-the-art algorithms. These results highlight MOSWO’s potential as a transformative tool for computational drug design. The flexibility and efficiency of MOSWO position it as a robust framework for addressing the challenges of modern drug therapy optimization. Future work could focus on hybrid techniques to boost its performance in complex or dynamic problem spaces, integrating pharmacokinetic and pharmacodynamic constraints and exploring its application in personalized medicine and combination therapy to further establish its versatility and scalability in clinical drug development.

## Figures and Tables

**Table 1:** IGD results of MOWSO compared to NSGA-II, MOEA/D, MOGWO, and MOPSO with respect to ZDT test instances.

Function	MOSWO	NSGA-II	MOEA/D	MOGWO	MOPSO
ZDT1	4.05 × 10^−3^	4.79 × 10^−3^	5.22 × 10^−3^	5.11 × 10^−3^	3.35 × 10^2^
ZDT2	3.99 × 10^−3^	4.92 × 10^−3^	5.28 × 10^−3^	1.20 × 10^−2^	1.86 × 10^−2^
ZDT3	5.52 × 10^−3^	5.47 × 10^−3^	8.02 × 10^−3^	5.46 × 10^−2^	1.01 × 10^−1^
ZDT4	4.22 × 10^−3^	4.57 × 10^−3^	5.42 × 10^−3^	1.60 × 10^−2^	2.67 × 10^−2^
ZDT6	3.40 × 10^−3^	3.69 × 10^−3^	4.22 × 10^−3^	4.34 × 10^−3^	4.46 × 10^−3^

**Table 2:** HV performance comparison of MOWSO, NSGA-II, MOEA/D, MOGWO, and MOPSO with respect to ZDT test instances.

Function	MOSWO	NSGA-II	MOEA/D	MOGWO	MOPSO
ZDT1	8.73 × 10^−1^	7.20 × 10^−1^	7.19 × 10^−1^	7.03 × 10^−1^	6.86 × 10^−1^
ZDT2	5.39 × 10^−1^	4.45 × 10^−1^	4.44 × 10^−1^	4.40 × 10^−1^	4.37 × 10^−1^
ZDT3	1.02 × 10^+0^	6.00 × 10^−1^	5.98 × 10^−1^	5.83 × 10^−1^	5.68 × 10^−1^
ZDT4	8.73 × 10^−1^	7.21 × 10^−1^	7.19 × 10^−1^	7.06 × 10^−1^	6.93 × 10^−1^
ZDT6	4.34 × 10^−1^	3.89 × 10^−1^	3.88 × 10^−1^	3.88 × 10^−1^	3.89 × 10^−1^

**Table 3:** Comparative analysis of spread results for MOWSO and other multiobjective algorithms with respect to ZDT test instances.

Function	MOSWO	NSGA-II	MOEA/D	MOGWO	MOPSO
ZDT1	1.67 × 10^−1^	4.05 × 10^−1^	5.89 × 10^−1^	9.34 × 10^−1^	1.46 × 10^+0^
ZDT2	1.71 × 10^−1^	4.30 × 10^−1^	6.41 × 10^−1^	8.06 × 10^−1^	1.18 × 10^+0^
ZDT3	3.57 × 10^−1^	4.31 × 10^−1^	8.86 × 10^−1^	1.10 × 10^+0^	1.77 × 10^+0^
ZDT4	2.56 × 10^−1^	4.47 × 10^−1^	4.96 × 10^−1^	9.35 × 10^−1^	1.42 × 10^+0^
ZDT6	4.74 × 10^−1^	4.17 × 10^−1^	6.72 × 10^−1^	4.54 × 10^−1^	4.92 × 10^−1^

**Table 4:** Evaluation of IGD metrics for MOWSO and competing algorithms with respect to DTLZ test instances.

Function	MOSWO	NSGA-II	MOEA/D	MOGWO	MOPSO
DTLZ1	1.85 × 10^−1^	2.69 × 10^−2^	6.91 × 10^−2^	4.85 × 10^−2^	2.78 × 10^−2^
DTLZ2	7.41 × 10^−2^	6.79 × 10^−2^	5.62 × 10^−2^	6.34 × 10^−2^	7.07 × 10^−2^
DTLZ3	2.05 × 10^+1^	6.80 × 10^−2^	2.17 × 10^−1^	1.44 × 10^−1^	7.11 × 10^−2^
DTLZ4	7.12 × 10^−2^	1.36 × 10^−1^	5.69 × 10^−2^	6.39 × 10^−2^	7.10 × 10^−2^
DTLZ5	4.81 × 10^−3^	5.68 × 10^−3^	3.19 × 10^−2^	1.92 × 10^−2^	6.46 × 10^−3^
DTLZ6	4.44 × 10^−3^	5.88 × 10^−3^	3.77 × 10^−2^	2.21 × 10^−2^	6.62 × 10^−3^
DTLZ7	1.00 × 10^−1^	1.09 × 10^−1^	9.40 × 10^−2^	8.86 × 10^−2^	8.31 × 10^−2^

**Table 5:** Comparative analysis of HV results for MOWSO and other multiobjective algorithms with respect to DTLZ test instances.

Function	MOSWO	NSGA-II	MOEA/D	MOGWO	MOPSO
DTLZ1	4.63 × 10^−1^	8.18 × 10^−1^	7.25 × 10^−1^	7.68 × 10^−1^	8.11 × 10^−1^
DTLZ2	6.77 × 10^−1^	5.30 × 10^−1^	5.53 × 10^−1^	5.40 × 10^−1^	5.28 × 10^−1^
DTLZ3	5.93 × 10^−2^	5.33 × 10^−1^	4.20 × 10^−1^	4.76 × 10^−1^	5.32 × 10^−1^
DTLZ4	6.96 × 10^−1^	4.95 × 10^−1^	5.52 × 10^−1^	5.40 × 10^−1^	5.29 × 10^−1^
DTLZ5	1.32 × 10^−1^	1.97 × 10^−1^	1.82 × 10^−1^	1.89 × 10^−1^	1.97 × 10^−1^
DTLZ6	1.32 × 10^−1^	1.97 × 10^−1^	1.80 × 10^−1^	1.88 × 10^−1^	1.97 × 10^−1^
DTLZ7	5.71 × 10^−1^	2.63 × 10^−1^	2.67 × 10^−1^	2.66 × 10^−1^	2.65 × 10^−1^

**Table 6:** Comparative analysis of spread results for MOWSO and other multiobjective algorithms with respect to DTLZ test instances.

Function	MOSWO	NSGA-II	MOEA/D	MOGWO	MOPSO
DTLZ1	7.16 × 10^−1^	5.03 × 10^−1^	1.30 × 10^+1^	8.83 × 10^−1^	4.69 × 10^−1^
DTLZ2	4.48 × 10^−1^	5.05 × 10^−1^	2.80 × 10^−1^	3.82 × 10^−1^	4.83 × 10^−1^
DTLZ3	1.03 × 10^+1^	6.68 × 10^−1^	1.58 × 10^+1^	1.04 × 10^+1^	4.98 × 10^−1^
DTLZ4	4.47 × 10^−1^	5.16 × 10^−1^	3.23 × 10^−1^	3.96 × 10^−1^	4.70 × 10^−1^
DTLZ5	2.06 × 10^−1^	4.64 × 10^−1^	1.67 × 10^−1^	3.25 × 10^−1^	4.82 × 10^−1^
DTLZ6	2.20 × 10^−1^	7.10 × 10^−1^	1.73 × 10^−1^	4.22 × 10^−1^	6.71 × 10^−1^
DTLZ7	5.70 × 10^−1^	4.92 × 10^−1^	1.31 × 10^+1^	9.25 × 10^−1^	5.44 × 10^−1^

**Table 7:** Sample of PK/PD data for testing MOSWO and other compared methods.

Patient ID	*Drug A Dose* (mg)	*Drug B Dose* (mg)	*C_max_* (ng/mL)	*T_max_* (h)	*AUC* (ng·h/mL)	Therapeutic Effect (%)	Toxicity Level (Grade)	Therapeutic Index (TI)
001	100	50	150	2	1200	85	1	1200
002	150	75	200	1.5	1500	90	2	750
003	200	100	250	2.5	1800	95	3	600
004	100	100	180	2	1300	80	1	1300
005	150	50	160	1.8	1400	88	2	700
006	200	75	220	2.2	1600	92	3	533
007	100	150	170	1.7	1250	82	1	1250
008	150	100	210	2.0	1550	89	2	775
009	200	50	190	2.3	1450	86	3	483
010	100	75	175	1.9	1350	84	1	1350
..	..	…	…	..	….	…	…	..
xxx	100	85	180	1.9	1550	81	1	1360

**Table 8:** Comparison of performance metrics of MOWSO with the other algorithms for the drug rehabilitation therapy design.

Algorithm	Dataset Size (*n*)	Pareto Front Coverage (%)	Hypervolume (10^3^)	Convergence (↓)	Diversity (↑)	Execution Time (s)
**MOSWO**	**10,000**	**86.7**	**3.94**	**0.012**	**0.88**	**50.0**
NSGA-II	10,000	85.2	3.75	0.015	0.85	45.0
MOEA/D	10,000	82.3	3.65	0.018	0.83	60.0
MOGWO	10,000	78.5	3.20	0.020	0.78	50.0
MOPSO	10,000	79.2	3.25	0.021	0.80	65.0

**Table 9:** Performance evaluation of MOSWO for cancer chemotherapy regimen optimization.

Algorithm	Dataset Size (n)	Pareto Front Coverage (%)	Hypervolume (10^3^)	Convergence (↓)	Diversity (↑)	Execution Time (s)
**MOWSO**	1000	**92.1**	**8.75**	**0.032**	**0.876**	187
NSGA-II	1000	85.3	7.92	0.045	0.831	215
MOEA/D	1000	87.6	8.31	0.039	0.812	193
MOGWO	1000	89.2	8.47	0.035	0.845	204
MOPSO	1000	82.7	7.64	0.048	0.793	176

**Table 10:** Performance comparison of MOSWO and other algorithms in drug design.

Metric	MOSWO	NSGA-II	MOEA/D	MOGWO	MOPSO
Therapeutic index (TI)	1.29 (Improved by 29%)	1.05	1.10	1.17	1.15
Toxicity level	0.25 (Reduced by 20%)	0.30	0.28	0.31	0.29
Cost efficiency	0.11 (Reduced by 9%)	0.23	0.29	0.26	0.31
Convergence rate (IGD)	0.0025	0.0030	0.0035	0.0035	0.0040
Hypervolume (HV)	0.83	0.75	0.78	0.78	0.76
Spread	0.15	0.20	0.18	9.21	0.25
Execution time (s)	125	150	140	150	160

## Data Availability

Data are contained within the article.
